# The directionality of anxiety and gaming disorder: An exploratory analysis of longitudinal data from an Australian youth population

**DOI:** 10.3389/fpsyt.2022.1043490

**Published:** 2022-11-04

**Authors:** Seungyeon Kim, Katrina E. Champion, Lauren A. Gardner, Maree Teesson, Nicola C. Newton, Sally M. Gainsbury

**Affiliations:** ^1^Brain and Mind Centre, School of Psychology, The University of Sydney, Sydney, NSW, Australia; ^2^The Matilda Centre for Research in Mental Health and Substance Use, The University of Sydney, Sydney, NSW, Australia

**Keywords:** problem gaming, gaming disorder, anxiety, longitudinal, adolescents, prevalence, pandemic

## Abstract

Gaming activities among adolescents have increased during the COVID-19 pandemic, bringing with it a growing concern for the potential harms of excessive gaming and its risk factors. Anxiety is frequently linked with gaming disorder, but studies investigating this association were mostly cross-sectional in design. Longitudinal studies that explore risk factors associated with gaming disorder are sparse and the trajectories of gaming disorder remain unclear. To address this paucity, the present study analyzed a large longitudinal dataset with a 12-month follow-up of 4,968 Australian adolescents (ages 13–14) during the pandemic. Logistic regression and multiple regression analyses were conducted to investigate the temporal relationships between anxiety, gaming frequency, the amount of money spent within video games, and gaming disorder. Prevalence rates for gaming disorder in adolescents aged 13 and 14 were 15 and 16%, respectively. The regression models indicated a bidirectional relationship between anxiety and gaming disorder symptoms, where higher levels of anxiety were associated with higher levels of gaming disorder 12 months later and vice versa. The study also found that the interaction between anxiety and higher gaming frequency could predict gaming disorder symptoms. Overall, the findings suggest that young adolescents may be more vulnerable to developing gaming disorder and highlight the importance of addressing the interactions between risk factors and gaming disorder in treatment approaches.

## Introduction

More than three-billion people worldwide report playing video games (1), with a large proportion of these players being aged under 18. In the United States, 71% of American youths (aged 13–17) play video games (2); in Australia, 78% of youths play video games (3). Youth populations are the most active in engaging with video games, playing on average for 106 min per day (3). Throughout the COVID-19 pandemic, the average time spent gaming has increased by approximately 28% (4–6), as video games provide not only leisure, but a platform to connect with others online. Research suggests that recreational screen time among adolescents has also increased during the pandemic by 6% (7). This has incited an urgency to investigate the risks associated with increased gaming, particularly among youth populations.

For a few individuals, gaming can become a persistent and harmful activity. The World Health Organization (WHO) defines “Gaming disorder” as repeated and persistent engagement with gaming (online or offline) over a 12-month period (8). It is characterized by an inability to control gaming (e.g., frequency, duration), the prioritization of gaming over other activities, and a persistence of gaming irrespective of the negative consequences. The recognition of gaming disorder and its harms by both the Diagnostic and Statistical Manual of Mental Disorders, 5th edition (DSM-5) and the International Classification of Diseases (ICD) 11th edition has facilitated greater research and clinical attention within the field (8, 9).

Variations in methodology (e.g., assessment tools), cultural and demographic factors across studies have made it difficult to establish the prevalence rates for gaming disorder. Nevertheless, adolescents are consistently reported to have a high prevalence of gaming disorder, with prevalence rates ranging from 2 to 14% (10–13). In a systematic review of 50 studies, Mihara and Higuchi reported that younger populations tended to have higher prevalence rates for gaming disorder than adults (10). A systematic review by Paulus et al. reported an average prevalence rate of 2% in children and adolescents and found that adolescents were particularly vulnerable to the negative effects of gaming disorder (11). Fam conducted a meta-analysis of 16 adolescent-focused studies and synthesized a 4.6% pooled prevalence estimate of gaming disorder among adolescents; however, differences in prevalence across age groups within adolescence were not investigated (12). Lastly, in a systematic review of 53 studies that utilized rigorous sampling criteria, Stevens et al. found that adolescents were more vulnerable to developing gaming disorder (13). The prevalence rate of gaming disorder in adolescents ranged from 9 to 14% across three studies that focused on samples aged between 12 and 15. Taken together, these findings suggest that younger populations—particularly young adolescents—may be more susceptible to developing gaming disorder, but the lack of studies that investigate populations of smaller age ranges makes it difficult to grasp whether different stages of adolescence affect gaming disorder.

Current theoretical models of gaming disorder suggest that individual vulnerabilities (e.g., psychopathology) may contribute to the development of maladaptive behaviors such as gaming disorder (14–16). The Interaction of Person-Affect-Cognition-Execution (I-PACE) model suggests that an interaction between vulnerability factors (e.g., psychopathology, personality traits) and affective or cognitive responses (e.g., impulsivity, coping styles, cognitive biases) contributes to the development and perpetuation of maladaptive behaviors (14, 15). Many studies have reported that gaming disorder is comorbid with mental health conditions such as depression and anxiety (17–20). However, most of these studies have utilized a cross-sectional design and thus, provide a limited understanding of how gaming disorder interacts with other mental health comorbidities over time.

Anxiety is associated with several maladaptive behavioral patterns such as problematic use of the Internet (21–23) and smartphones (24, 25). Anxiety is also associated with problematic gambling, where individuals who experience problematic gambling are more likely to report anxiety symptoms than those who do not (26, 27). González-Bueso et al. (17) reviewed 24 studies on gaming disorder and found that 17 studies reported an association between gaming disorder and anxiety, whereby the presence of one increased the likelihood of the other. Moreover, the severity of gaming disorder was often reported to be associated with the severity of anxiety in several cross-sectional survey studies (19, 28–30).

With reference to the I-PACE model, an individual may respond to anxiety by playing video games, and subsequently learn that gaming is an effective way to elevate mood states or avoid negative mood states (14, 15). Avoidance behaviors play a significant role in maintaining anxiety symptomology (31). The immediate gratification—the relief of negative mood states—brought by gaming may encourage gaming behaviors to recur in further situations that elicit negative affect. The repetition of this behavioral pattern may ultimately lead to habit formation and, as a result, gaming may become prioritized over other important aspects of life (14, 15). The theoretical framework of the I-PACE model suggests a directional relationship between anxiety and gaming disorder; one where anxiety influences the development and maintenance of gaming disorder, but this is difficult to observe with cross-sectional data alone.

Another factor associated with gaming disorder is gaming intensity. The present study operationalizes gaming intensity as gaming frequency and expenditure within video games. Gaming frequency—how often one engages with video games—is often reported to be associated with gaming disorder (32–34). Longer and more frequent gaming sessions may indicate an impaired ability to control gaming behaviors. Although lesser understood, the amount of money an individual spends within a video game may also be indicative of gaming severity and potentially gaming disorder.

Many video games offer virtual content within the game that can be purchased using real-world currency. These range from cosmetic items that can change the appearance of game characters or weapons, to items that provide a competitive advantage to the player, to subscriptions that allow players to access game content. Among these are loot boxes—a virtual item that provides players a chance to randomly obtain an in-game item of varying rarity (35, 36). Due to their resemblance to gambling, loot boxes have garnered much interest from both media and research. In research, purchasing loot boxes has often been reported to be positively correlated with gaming disorder (35–38); that is, individuals experiencing severe gaming disorder were likely to spend more on loot boxes. However, these studies were often cross-sectional in design, leaving the trajectory of the relationship between loot box purchases and gaming disorder unknown. The association between gaming expenditure and gaming disorder has also received little attention in literature, much less in conjunction with the risk factors associated with gaming disorder. Thus, it is difficult to know how gaming expenditure interacts with gaming disorder, and whether this is affected by other risk factors (e.g., anxiety).

Due to the COVID-19 pandemic, the average time youth spend gaming, gaming disorder, and anxiety levels have increased (39–42). Yet, the questions of who is vulnerable and how these risk factors interact with each other remain unclear. Although there are longitudinal studies that investigate less specific internet use disorder and mental health comorbidities (43, 44), the number of longitudinal studies that investigate anxiety and gaming disorder is scarce and often conducted with small or non-representative samples (45, 46). To address this paucity, the present study analyzed longitudinal data that assessed various health outcomes among a large cohort of Australian adolescents (aged 13–14) over 12 months, within the context of the COVID-19 pandemic. The aims of this investigation were fourfold: (1) to ascertain the prevalence and correlates of gaming disorder in a large national sample of young adolescents; (2) to evaluate the directionality between anxiety and gaming disorder; (3) to evaluate the directionalities of gaming frequency, video game expenditure, and gaming disorder; and (4), to evaluate the temporal interactions between all these variables.

Given the current understanding of anxiety, gaming disorder, and gaming intensity, the present study conducted an exploratory analysis with the following hypotheses:

**H_1_**: Higher levels of anxiety will predict both an increase in gaming disorder symptoms and a greater likelihood of a gaming disorder diagnosis after 12 months.

**H_2_**: Gaming disorder symptomology will predict an increase in anxiety levels after 12 months.

**H_3_**: Gaming intensity (gaming frequency and video game expenditure) will predict an increase in gaming disorder symptoms after 12 months.

**H_4_**: Greater gaming intensity and higher anxiety levels will predict an increase in gaming disorder symptomology and a gaming disorder diagnosis after 12 months.

## Materials and methods

### Sample and design

The longitudinal data used in this analysis were drawn from the Health4Life Initiative—a cluster randomized controlled trial following a cohort of 6,640 students (baseline M_age_ = 12.6, *SD* = 0.5; 48% female), across 71 Australian schools in the three Australian states of New South Wales, Western Australia, and Queensland, from 2019 to 2022 (47). All students completed online self-report surveys in a classroom setting. This study uses data on anxiety, gaming disorder, and gaming intensity (e.g., gaming frequency and spending in video games) collected at 12-months (2021; M_age_ = 13.7) and 24-months (2022; M_age_ = 14.7) post-baseline. The follow-up rates for the Health4Life study were 83% at 12 months (*N* = 4,968), and 75% at 24 months (*N* = 4,466). Full details of the study protocol are published elsewhere (47).

### Measures

#### Anxiety

The Patient-Reported Outcome Measurement Information System (PROMIS) pediatric item bank (48) is an instrument used to assess various health-related domains for children and adolescents, including anxiety and depression. Anxiety was assessed using the anxiety scale (PROMIS-A). PROMIS-A is a 13-item instrument where children are prompted to rate how frequently they experienced anxiety symptoms in the past 7 days on a 5-point Likert scale that ranges from “Never” to “Almost always.” Total scores range from 13 to 65, where higher scores denote greater severity of anxiety. The PROMIS-A had a Cronbach’s alpha of 0.94–0.96.

#### Gaming disorder

Gaming disorder was measured using the short version of the Internet Gaming Disorder (IGD) Scale (49). The IGD scale consists of nine items (yes–no) regarding gaming behaviors that correlate to the nine criteria for IGD provided by the DSM-5 (preoccupation, tolerance, withdrawal, persistence, escape, problems, deception, displacement, and conflict). Total scores range from 0 to 9, where higher scores indicate greater severity for problematic gaming behaviors. Total scores of 5 or greater met the criteria for a diagnosis of IGD. The scale had a Cronbach’s alpha of 0.83–0.84.

#### Gaming intensity

Gaming frequency (“In a typical month, how often do you play video games?”) and gaming expenditure (“In a typical month, what is the total dollar amount you spend within video games on things like loot-boxes, in-app purchases, skins, in-game currency, or subscriptions?”) were used to measure gaming intensity. The categories for gaming frequency included “Not at all,” “1–3 times per month,” “Once a week,” “Several times a week,” and “Daily.”

### Statistical analyses

Gaming disorder outcomes were divided into two variables: a gaming disorder symptoms variable which analyzed the cumulative score of the gaming disorder scale as a continuous variable, and a gaming disorder diagnosis variable, which measured whether the respondent met the criteria for a diagnosis of gaming disorder. The latter was dichotomized into 0 = “no diagnosis” and 1 = “gaming disorder diagnosis.” Gaming frequency was categorized as 0 = “Not at all,” 1 = “1–3 times per month,” 2 = “Once a week,” 3 = “Several times a week,” and 4 = “Daily.”

The statistical analyses consisted of a bivariate correlation—to measure the linear relationships between the anxiety, gaming disorder, and gaming intensity variables. A one-way ANOVA was conducted to analyze differences in the means of gaming frequency and anxiety scores, gaming disorder scores, and gaming expenditure. All statistical analyses, including assumption testing, were conducted using the software SPSS, version 28 (50).

Logistic regression was used to investigate whether anxiety, gaming frequency and gaming disorder symptoms at age 13 (12-month data) could predict a diagnosis of gaming disorder 12 months later at age 14 (24-month data). Multiple linear regression analyses were conducted using anxiety at age 13 (Model 1) and gaming frequency at age 13 (Model 2) as independent variables to predict gaming disorder symptoms at age 14. Model 3 calculated both anxiety levels and gaming frequency at age 13 as independent variables to predict gaming disorder symptoms at age 14. Model 4 analyzed gaming disorder symptoms at age 13 as a predictor of anxiety levels 12 months later at age 14.

## Results

### Descriptive statistics and correlations

The descriptive statistics and bivariate correlations of all variables of interest are presented in Tables 1, 2, respectively. At age 13, 15% of respondents (*N* = 697) met the criteria for gaming disorder (95% CI [13.9, 15.9]), and 16% of respondents (*N* = 596) met the criteria for gaming disorder at age 14 (95% CI [14.7, 16.7]).

**TABLE 1:** Descriptive statistics across all independent and dependent variables.

Variable	*N*	Mean	*SD*
Anxiety (age 13)	4,968	24.05	12.29
Anxiety (age 14)	4,466	24.85	12.72
Gaming disorder symptoms (age 13)	3,600	2.46	2.49
Gaming disorder symptoms (age 14)	3,171	2.47	2.52
Gaming disorder diagnosis (age 13)	4,678	–	–
Gaming disorder diagnosis (age 14)	4,384	–	–
Gaming frequency (age 13)	4,809	–	–
Gaming expenditure (age 13)	3,408	17.74	185.31

Gaming disorder diagnosis and gaming frequency are categorical variables. Unit for gaming expenditure = AUD.

**TABLE 2:** Pearson correlation matrix for all analyzed variables.

	1	2	3	4	5	6	7	8
1. Anxiety (age 13)	1.00	0.50	0.15**	0.12**	0.12**	0.09**	–0.01	–0.03
2. Anxiety (age 14)	0.50**	1.00	0.10**	0.18**	0.09**	0.14**	−0.06**	0.01
3. Gaming disorder symptoms (age 13)	0.15**	0.10**	1.00	0.46**	0.82**	0.38**	0.35**	0.10**
4. Gaming disorder symptoms (age 14)	0.12**	0.18**	0.46**	1.00	0.38**	0.82**	0.20**	0.03
5. Gaming disorder diagnosis (age 13)	0.12**	0.09**	0.82**	0.38**	1.00	0.36**	0.34**	0.09**
6. Gaming disorder diagnosis (age 14)	0.09**	0.14**	0.38**	0.82**	0.36**	1.00	0.20**	0.02
7. Gaming frequency (age 13)	–	–	–	–	0.34**	0.20**	–	–
8. Gaming expenditure (age 13)	–0.03	0.01	0.10**	0.03	0.09**	0.02	0.06**	1.00

**p > 0.01.

Anxiety levels at age 13 showed a significant positive correlation with gaming disorder symptoms at age 13 (*r* = 0.17, *p* < 0.01) and age 14 (*r* = 0.12, *p* < 0.01); that is, higher anxiety levels were correlated with greater gaming disorder symptoms. Similarly, higher anxiety levels were significantly correlated with a gaming disorder diagnosis at age 13 (*r* = 0.14, *p* < 0.01) and at age 14 (*r* = 0.11, *p* < 0.01). Gaming frequency displayed a significant positive correlation with a diagnosis of gaming disorder at age 13 (*r* = 0.28, *p* < 0.01) and at age 14 (*r* = 0.12, *p* < 0.01), indicating that more frequent gaming was associated with greater gaming disorder symptoms and a diagnosis of gaming disorder. Greater gaming expenditure was significantly correlated with increased gaming frequency (*r* = 0.06, *p* < 0.01), greater gaming disorder symptoms at age 13 (*r* = 0.10, *p* < 0.01) and a gaming disorder diagnosis at age 13 (*r* = 0.09, *p* < 0.01); however, there was no significant correlation between gaming expenditure and anxiety, gaming disorder symptoms, nor a diagnosis of gaming disorder at age 14.

A one-way ANOVA demonstrated statistically significant differences between gaming frequency and anxiety levels at age 13 [*F*(4, 4,628) = 7.42, *p* ≤ 0.001] and age 14 [*F*(4, 3,663) = 8.42, *p* ≤ 0.001] and gaming frequency and gaming disorder symptoms at age 13 [*F*(4, 3,585) = 166.23, *p* ≤ 0.001] and age 14 [*F*(4, 2,598) = 35.22, *p* ≤ 0.001]. Moreover, a Tukey *post-hoc* test revealed statistically significant differences in gaming disorder symptoms (*p* = 0.05) between adolescents who played video games daily and adolescents who played video games several times a week.

### Regression analyses

As there was no linear relationship observed between gaming expenditure and anxiety, gaming disorder symptoms, or a diagnosis of gaming disorder, gaming expenditure was excluded from the regression analyses.

A logistic regression analysis was conducted to determine the extent to which anxiety levels at age 13 and gaming frequency at age 13 could predict a gaming disorder diagnosis at age 14 (see Table 3). The overall model was statistically significant [χ2 (6) = 845.53, *p* < 0.001], accounted for 25% of variance in whether a diagnosis of gaming disorder was present at age 14, and correctly predicted 86.5% of cases. Anxiety levels at age 13 (*p* < 0.001), gaming disorder levels at age 13 (*p* < 0.001), gaming several times a week (*p* < 0.001) and gaming daily (*p* < 0.001) were significant predictors of a gaming disorder diagnosis at age 14. As anxiety levels and the number of gaming disorder symptoms increased, the likelihood of having a diagnosis of gaming disorder after 12 months increased by 1.01- and 1.50-fold, respectively. Adolescents who played games several times a week or daily were 1.88 times and 1.44 times more likely to have a diagnosis of gaming disorder after 12 months. Gaming 1–3 times per week or once a week did not significantly predict a diagnosis of gaming disorder.

**TABLE 3:** Logistic regression analysis of anxiety levels (age 13), gaming frequency (age 13), gaming disorder symptoms (age 13), and gaming disorder diagnosis (diagnosed vs. not diagnosed) at age 14.

Independent variables	B (SE)	Wald	Odds ratio	95% CI
1. Anxiety (age 13)	0.01 (0.03)	7.52**	1.01	(1.00, 1.02)
2. Gaming disorder symptoms (age 13)	0.40 (0.02)	551.18**	1.50	(1.45, 1.55)
3. Gaming frequency (not at all)	–	43.70	–	–
4. Gaming frequency (1–3 times per month)	−0.17 (0.20)	0.70	0.85	(0.57, 1.25)
5. Gaming frequency (once a week)	−0.02 (0.16)	0.01	0.99	(0.72, 1.35)
6. Gaming frequency (several times a week)	0.63 (0.13)	24.06**	1.88	(1.46, 2.42)
7. Gaming frequency (daily)	0.37 (0.14)	6.97**	1.44	(1.10, 1.89)

**p < 0.01; Nagelkerke *R*^2^ = 0.25; 95% CI. Gaming frequency (not at all) was used as the reference point for subsequent categories of gaming frequency.

Assumption tests indicated that multicollinearity was not a concern for variables used to predict gaming disorder symptoms (Anxiety, Tolerance = 0.99, VIF = 1.01; Gaming frequency [Not at all], Tolerance = 0.57, VIF = 1.75; Gaming frequency [1–3 times per month], Tolerance = 0.67, VIF = 1.50; Gaming frequency [Once a week], Tolerance = 0.62, VIF = 1.62; Gaming frequency [Several times a week], Tolerance = 0.54, VIF = 1.87; Gaming frequency [Daily], Tolerance = 0.60, VIF = 1.66). The data also met the assumption of independent errors (Durbin-Watson value = 1.97). A scatterplot of standardized residuals showed that the data met the assumptions of homoscedasticity and a normal P-Plot of standardized residuals showed that data points were close to the line, indicating that the errors were approximately normally distributed.

The multiple regression models analyzing the influence of anxiety levels, gaming disorder symptoms, and gaming frequency at age 13 on gaming disorder symptoms at age 14 are presented in Table 4. Model 1 analyzed anxiety levels at age 13. The results indicated that anxiety levels at age 13 had a statistically significant influence on gaming disorder symptoms at age 14. Gaming frequency at age 13 was also found to influence gaming disorder symptoms at age 14 (Models 2 and 3; see Table 4), where more frequent levels of gaming (e.g., gaming daily) significantly predicted a slight increase in gaming disorder symptoms 12 months later. Less frequent levels of gaming (e.g., not gaming at all, gaming 1–3 times per month, or once a week) predicted a decrease in gaming disorder symptoms 12 months later. When both anxiety levels and gaming frequency were analyzed together (Model 3), the model significantly predicted a change in gaming disorder symptoms 12 months later. However, the predictive value of anxiety levels and gaming daily decreased in comparison to Models 1 and 2. Gaming several times a week did not significantly predict gaming disorder symptoms after 12 months. Lastly, a regression model was conducted to analyze the influence of gaming disorder symptoms at age 13 on anxiety levels at age 14 (Model 4; see Table 5). Results indicated that gaming disorder symptoms had a significant influence on anxiety levels, where a one-unit increase in gaming disorder symptoms at age 13 predicted a 0.54 unit increase in anxiety levels 12 months later.

**TABLE 4:** Multiple linear regression analyses using anxiety levels (age 13) and gaming frequency (age 13) to predict gaming disorder symptoms (age 14).

	Gaming disorder symptoms at age 14					
	Model 1		Model 2		Model 3	
Variables	B	95% CI	B	95% CI	B	95% CI
Anxiety (age 13)	0.03***	(0.02, 0.03)			0.02***	(0.01, 0.02)
Gaming frequency (not at all)			−0.78***	(−0.96, −0.59)	−0.79***	(−0.97, −0.60)
Gaming frequency (1–3 times per month)			−0.93***	(−1.1, −0.72)	−0.95***	(−1.16, −0.74)
Gaming frequency (once a week)			−0.55***	(−0.75, −0.35)	−0.68***	(−0.88, −0.48)
Gaming frequency (several times a week)			0.17	(−0.01, 0.35)	0.04	(−0.14, 0.21)
Gaming frequency (daily)			0.53***	(0.34, 0.73)	0.32***	(0.13, 0.51)

*R*^2^_adj_ for Model 1 = 0.02; *R*^2^_adj_ for Model 2 = 0.06; *R*^2^_adj_ for Model 3 = 0.07. *** *p* < 0.001. Gaming frequency (not at all) was used as the reference for categories within gaming frequency.

**TABLE 5:** Linear regression analysis using gaming disorder symptoms (age 13) to predict anxiety levels 12 months later (age 14).

	Anxiety levels at age 14	
	Model 4	
Variables	B	95% CI
Gaming disorder symptoms (age 13)	0.54***	(0.34, 0.73)

*R*^2^_adj_ for Model 4 = 0.01. ***p < 0.001.

## Discussion

This study aimed to ascertain the prevalence rates of gaming disorder on a nationwide sample of young adolescents aged 13–14 and explore the effects of anxiety and gaming intensity (gaming frequency and spending money within video games) on both gaming disorder symptomology and a gaming disorder diagnosis after 12 months. This study also analyzed the effects of gaming disorder symptomology on anxiety levels after 12 months. This study analyzed data from the largest longitudinal study on adolescents aged 13–14 to date.

Of note, the 15% [95% CI (13.9, 15.9)] and 16% [95% CI (14.7, 16.7)] prevalence rates of gaming disorder in adolescents (aged 13 and 14, respectively) found in this study was much higher than the 2–4.6% reported in previous studies (10–13). Studies that reported the prevalence of gaming disorder in adolescents often investigated samples with wider age ranges, usually between 12 and 18 years (12, 13). The few studies that focused on a smaller age range in young adolescents (e.g., 12–15) found overall higher prevalence rates of gaming disorder (between 9 and 14%) (43, 51, 52), which were closer to what this study reports. Even so, these past studies investigated a non-representative sample or had relatively smaller sample sizes than the present study, which analyzed a much larger cohort (*N* = 4,968) of young adolescents over 12 months.

Adolescence is an important period of transition marked by rapid changes in maturity, growth, and independence. When using a broad age range to investigate adolescent populations, it is difficult to control for factors that may differentially influence stages of adolescence. The present study focused on adolescents aged 13–14. The high prevalence rates of gaming disorder within this age range suggest that factors specific to this stage of early adolescence (e.g., puberty, starting secondary education, increased autonomy, and increased exposure to online technologies) may influence the risk of adolescents developing gaming disorder. More research is needed to understand how these factors interact with video gaming and gaming disorder. These prevalence findings also emphasize the importance of addressing the unique experiences of young adolescence in treatment approaches for gaming disorder in youths.

The results of the bivariate correlation suggest that anxiety levels, gaming disorder symptomology (at age 13 and age 14), and a diagnosis of gaming disorder (at age 13 and 14) were positively correlated; that is, higher anxiety levels were associated with greater gaming disorder symptomology and the presence of a gaming disorder diagnosis across the span of 12 months. This aligns with extant findings of cross-sectional studies, where individuals who experienced gaming disorder symptoms, or had a diagnosis of gaming disorder, also experienced higher levels of anxiety (19, 29).

The results of the regression analyses support the first hypothesis which states that higher levels of anxiety would predict an increase in gaming disorder symptoms after 12 months and a greater likelihood of a gaming disorder diagnosis after 12 months (see Tables 3, 5). These findings can be interpreted using the theoretical framework of the I-PACE model. The I-PACE model suggests that an individual who is experiencing high levels of anxiety may seek to resolve negative affect by playing video games (14, 15). The result of this is habit formation: the individual learns the association between gaming and mood regulation and thus, continues to play video games to relieve negative mood states. This may subsequently lead to the development of distorted cognitions of gaming such that gaming becomes prioritized over other important aspects of life. It is also of note that this behavioral pattern is more likely to occur within the context of the COVID-19 pandemic, with literature showing an increase in levels of anxiety and greater engagement with video games in adolescents (4–7, 40–42).

In support of hypothesis two, an increase in gaming disorder symptoms was found to predict higher levels of anxiety after 12 months (Table 5). Although individuals may initially engage with video games to manage high anxiety levels (29, 53, 54), the presence of gaming disorder symptoms appears to exacerbate anxiety levels over time. This is supported by past research that investigated the associations between gaming disorder symptomology and affective disorders (17, 18, 55–57). Although existing studies have provided cross-sectional evidence, the present study demonstrates the temporal relationship between gaming disorder symptoms and anxiety. These findings suggest that a bidirectional relationship exists between anxiety and gaming disorder: anxiety may be a catalyst for the development and perpetuation of gaming disorder, and in turn, gaming disorder symptoms may perpetuate high levels of anxiety.

Hypothesis three—that gaming intensity would predict an increase in gaming disorder symptoms in later stages—was only partially supported by these findings (Tables 3, 4). The logistic regression analysis showed that playing video games several times a week or daily was associated with a diagnosis of gaming disorder after 12 months (see Table 3). Playing video games daily was also shown to be associated with an increase in gaming disorder symptoms after 12 months (see Table 4). This aligns with past findings which suggest that frequently playing video games is associated with gaming disorder and that higher gaming frequencies may be indicative of an inability to self-regulate gaming (32–34).

Our findings indicate that spending money within video games was associated with gaming frequency, gaming disorder symptoms and diagnosis within the same timepoint. Adolescents experiencing gaming disorder were likely to spend more money within video games than adolescents without gaming disorder. Past research also found a positive association between spending within video games—specifically, loot box spending—and gaming disorder (37, 38, 58, 59). However, the amount of money spent within video games was not associated with whether adolescents experienced problematic gaming over time; that is, gaming expenditure at age 13 did not significantly predict a diagnosis of gaming disorder in adolescents aged 14. Our findings indicate that gaming expenditure may not be correlated with the development of gaming problems for young adolescents. There are two possible explanations for this. First, participants were young (13–14) and thus, may have had limited access to funds. Or second, it is possible that gaming disorder predicts gaming expenditure instead. That is, adolescents experiencing gaming disorder may tend to spend more within video games over time than adolescents without gaming disorder.

Hypothesis four stated that greater gaming intensity and anxiety levels would predict an increase in gaming disorder symptoms and a diagnosis in the future. This hypothesis was partially supported (see Table 5). When anxiety levels and gaming frequency were taken together to predict gaming disorder symptoms, the overall predictive value of the model increased. This suggests that adolescents with high levels of anxiety who engage with video games daily, may be more susceptible to developing a diagnosis of gaming disorder (or an increase in gaming disorder symptoms). It is the interaction of anxiety and gaming daily that influence the development of gaming disorder. This is further explained by the I-PACE model: that engaging with video games to alleviate negative affect (e.g., anxiety) results in the development of maladaptive coping strategies (e.g., gaming disorder symptoms), distorting cognitions about gaming (e.g., that gaming will alleviate negative affect) and ultimately increasing gaming behaviors (e.g., more frequent gaming). Treatment for gaming disorder should then seek to address this cycle of maladaptive coping and cognitions.

### Strengths and limitations

This study had several limitations. Despite yielding statistically significant results, the regression models explained overall low levels of variance. The models that solely utilized anxiety and gaming frequency as predictors of gaming disorder accounted for < 5% variation. This implies that anxiety and gaming frequency, as standalone variables, only weakly explain the increase in gaming disorder symptoms after 12 months. As a result, addressing anxiety or gaming frequency alone when supporting youths with gaming disorder may not be effective as an intervention strategy. Rather, the interaction between anxiety and gaming frequency must be addressed.

Anxiety was chosen as the focus of this study for its association with problematic behaviors and maladaptive coping strategies (e.g., avoidant behaviors), but a broader exploration of affect and cognitions may further clarify the underlying mechanisms of gaming disorder. It is likely that other risk factors for gaming disorder, such as depression (17, 52), and the interactions between these risk factors, may provide predictive and directional insight into the development or exacerbation of gaming disorder. Thus, future research investigating gaming disorder in young adolescents should consider how the interactions of other risk factors (e.g., depression) may affect gaming disorder.

This study operationalized gaming intensity as gaming frequency and gaming expenditure and did not examine how the duration of playing time may have affected gaming disorder symptoms or a gaming disorder diagnosis after 12 months. Alongside gaming frequency, research has shown that the duration of playing time is associated with gaming disorder; specifically, longer playing times are associated with harmful levels of gaming (33, 34). This study utilized data from the Health4Life Initiative, a cluster randomized controlled trial of an eHealth intervention wherein gaming was not a primary or secondary outcome of interest in the trial (47). Thus, playing time was not measured and is an area for future research to consider.

The measure for gaming expenditure in the data did not discern between the specific in-game content that was purchased. Thus, this study does not consider the influences of specific mechanisms of in-game purchases on gaming expenditure and gaming disorder symptoms. For instance, loot box purchases may be associated with greater spending due to the similarities with gambling machines (58, 59). Research also suggests that the structural features of in-game purchases have different levels of associated risk (60), so differentiating between in-game purchases may be important for understanding spending behaviors and gaming disorder over time. Nevertheless, the present study provides insight into the directionality of spending and gaming disorder—that gaming disorder may influence spending behaviors in adolescents.

A key feature of this study is that it investigates gaming disorder symptoms in a large cohort of Australian youths aged 13–14 over a 12-month period. The population comprised of a relatively small proportion of youths who had clinical-level symptoms for anxiety and gaming disorder. As a result, our analyses provide crucial insight into the trajectory of gaming behaviors and gaming disorder symptoms in young adolescents who may have yet to develop any gaming problems. Additionally, the data used in this analysis were collected within the context of the COVID-19 pandemic—a period wherein emerging research has reported increases in engagement with video games and screen usage, and anxiety levels among youths (4–7, 40–42). This study captures the impact of the pandemic on adolescents and identify vulnerabilities within this population.

## Conclusion

This study aimed to determine the prevalence of gaming disorder and understand the temporal and directional relationships between anxiety and gaming disorder. This study analyzed longitudinal data of a large Australian youth population over a 12-month period. The high prevalence rates of gaming disorder in adolescents aged 13 and 14 indicate that factors unique to this period of early adolescence influences the vulnerability of youths developing gaming disorder. This finding highlights a need for future research and treatment approaches to focus on early adolescence. This study also implicates a bidirectional relationship between anxiety and gaming disorder symptoms, whereby higher levels of anxiety predict an increase in gaming disorder symptoms and vice versa. Gaming frequency was also shown to be associated with an increase in gaming disorder symptoms after 12 months. However, when analyzed separately, anxiety and gaming frequency were weak predictors of gaming disorder. Instead, the interaction between anxiety and gaming frequency was shown to be a better predictor of gaming disorder symptoms after 12 months. Lastly, this study found that spending within video games did not predict an increase in gaming disorder symptoms in young adolescents.

Given the increase in time spent gaming and mental health problems experienced by youths throughout the COVID-19 pandemic (5, 40–42), this study provides the early steps in identifying vulnerable populations and understanding how risk factors interact to influence the development or exacerbation of gaming disorder. Further research and treatment approaches should investigate the interactions between risk factors associated with gaming disorder, and how these interactions increase gaming disorder symptoms.

## Data availability statement

The raw data supporting the conclusions of this article will be made available by the authors, without undue reservation.

## Ethics statement

The studies involving human participants were reviewed and approved by the Human Research Ethics Committees, University of Sydney (2018/882). Written informed consent to participate in this study was provided by the participants’ legal guardian/next of kin.

## Author contributions

SK conceptualized the study, conducted the data analysis and interpretation, and drafted the manuscript. KC, LG, MT, and NN acquired the data. MT, NN, and KC secured funding for the study. SG contributed expertise to the conceptualization of the study and interpretation. All authors contributed expertise, critically revised, and approved the final version of the manuscript and agreed to be accountable for all aspects of the work.
